# Speech-Controlled Reconfigurable Intelligent Metasurface for Real-Time Wireless Power Transfer and Communication

**DOI:** 10.34133/research.0831

**Published:** 2025-08-12

**Authors:** Lin Dong, Liming Si, Yueze Liu, Qitao Shen, Pengcheng Tang, Genhao Wu, Rong Niu, Qingqing Wu, Weiren Zhu

**Affiliations:** ^1^ Beijing Key Laboratory of Millimeter Wave and Terahertz Technology, School of Integrated Circuits and Electronics, Beijing Institute of Technology, Beijing 100081, China.; ^2^ State Key Laboratory of Environment Characteristics and Effects for Near-Space, Beijing Institute of Technology, Beijing 100081, China.; ^3^ School of Mechanical Engineering, Beijing Institute of Technology, Beijing 100087, China.; ^4^ Department of Electronic Engineering, Shanghai Jiao Tong University, Shanghai 200240, China.

## Abstract

As the Internet of Things (IoT) continues to evolve, a growing number of wireless sensors have been integrated into daily life, posing new challenges to energy supply and communication. Point-to-point dynamic wireless power transfer and communication, enabled by user-based positioning and tracking services, hold great potential in the IoT. Here, we propose a speech-controlled reconfigurable intelligent metasurface (RIS) that translates natural-language commands into dynamically shaped electromagnetic beams by combining speech interaction, low-power RIS control, and a template matching algorithm, supporting real-time data communication and wireless power transfer for both static and moving targets. Unlike conventional RISs that rely on pre-defined control and external processing units, our approach provides the metasurface with visual and linguistic perception capabilities, enabling a paradigm shift from passive reconfiguration to active multimodal intelligence. The experimental results confirm the effectiveness of the speech-controlled RIS, while the measured results demonstrate that the RIS can provide a stable dc output that exceeds 4.61 V on dynamic targets. By enabling intuitive human–device interaction and effectively meeting the power supply requirements of small sensors, the proposed concept demonstrates strong application potential in IoT scenarios.

## Introduction

With the rapid advancement of wireless communication, the Internet of Things (IoT) has become deeply integrated into daily life, substantially transforming the way that people interact with their environments [1–3]. However, the explosive proliferation of wireless sensors poses new challenges to both energy supply and communication. Traditional wireless sensors are typically powered by batteries and experience usage interruptions due to battery depletion [4]. In addition, their broadcast communication exacerbates communication congestion in dense networks [5]. The surge of unmanned platforms (unmanned aerial vehicles, mobile robots, and autonomous vehicles) has introduced a large number of fast-moving nodes. Their continuous motion further strains the batteries and overcrowds the shared channels. Hence, intelligent wireless power transfer (WPT) and point-to-point communication, enabled by user-based positioning and tracking services, offer great potential for the successful realization of IoT systems [6,7].

Metasurfaces have attracted significant interest from researchers due to their extraordinary capacity to manipulate the amplitudes [8–10], phases [11–14], and polarizations [15–19] of electromagnetic (EM) waves. A series of intriguing applications of metasurfaces have been explored recently, including EM absorption [20–22], vortex beam generation [23–27], holography [28–30], near-field focusing (NFF) [31–33], and WPT [34–36]. To achieve dynamic wave front modulation, programmable metasurfaces (PMSs) based on digitally programmable meta-atoms have been proposed [37–40]. PMSs exhibit advantages such as a lower cost, reduced power consumption, and simpler control circuits, making them suitable for large-scale deployments, energy-constrained environments, and point-to-point directional WPT applications [41,42]. However, most applications of PMSs have relied on manual control, primarily focusing on the validation of pre-defined functionalities, thereby limiting their adaptability and flexibility in dynamic environments. A reconfigurable intelligent metasurface (RIS) is an enhanced PMS that integrates intelligent algorithms to automatically meet user demands and adapt to ever-changing environments. Several RISs have been developed to achieve self-adaptive beam steering, cloaking, dynamic response, frequency recognition, and speech recognition [43–50].

Currently, RISs enabled by user-based positioning and tracking services to achieve point-to-point dynamic WPT and communication can be divided into EM-wave-based wireless positioning, sensor-based positioning, and vision-based positioning. Although EM-wave-based wireless positioning offers the advantage of being unaffected by light and weather conditions, it is costly and complex to achieve real-time and self-adaptive EM responses in complex environments [51]. While sensor-based positioning provides accurate localization, it requires additional modules at the receiver side, increasing system complexity and hardware overhead [52]. In contrast, vision-based positioning employs low-cost, widely available cameras to achieve subcentimeter spatial resolution and deliver rich multimodal information (e.g., shape, pose, and gesture) without introducing additional radio frequency (RF) hardware or spectrum consumption. However, deep-learning-based object detectors such as You Only Look Once (YOLO), while offering superior accuracy and robustness, demand high computational resources and large-scale labeled datasets for training [53,54]. In many existing systems, RIS interaction remains nonintuitive, often requiring manual configuration and lacking responsiveness to changing user demands. Most prototypes still depend on external personal computers with a large physical size and high power consumption, hindering portable and energy-efficient deployment in IoT scenarios. Moreover, the computational resources consumed by vision-based detection and speech processing in WPT–RIS systems limit their scalability toward integrated multimodal control, such as concurrent speech recognition, target tracking, and EM wave manipulation [51–54]. Therefore, RIS requires higher hardware integration, faster response speeds, more convenient interaction methods, and a multimodal operation capability that integrates vision perception, speech recognition, and EM control to meet the growing demands of IoT systems for user positioning, tracking, WPT, and a wide range of applications.

In this paper, we propose a speech-controlled reconfigurable intelligent metasurface (SC-RIS) to achieve point-to-point dynamic WPT and communication simultaneously. By integrating speech interaction, low-power RIS control, and a template matching algorithm, the proposed SC-RIS establishes a self-adaptive closed-loop control system that operates in speech-commanded mode, enabling real-time, precise data communication and efficient WPT to both static and dynamic targets. In contrast to conventional RIS architectures that depend on pre-defined control strategies and external processing units, the proposed approach provides the metasurface with integrated visual and linguistic perception capabilities, thereby enabling a transition from passive reconfiguration to actively adaptive, multimodal EM control. Experimental results confirm that the SC-RIS enables selective wireless charging and information transmission to static and dynamic targets based on speech commands, providing users with a natural and intuitive way to interact with devices. Meanwhile, the power of the target was measured, demonstrating that the SC-RIS can provide a stable direct current (dc) output exceeding 4.61 V on dynamic targets, effectively meeting the power supply requirements of small sensors. The proposed concept exhibits considerable application potential in the IoT, including sensors, small mobile devices, smart home systems, and unmanned aerial vehicles.

## Results and Discussion

### Theory and design

**Fig. 1.**
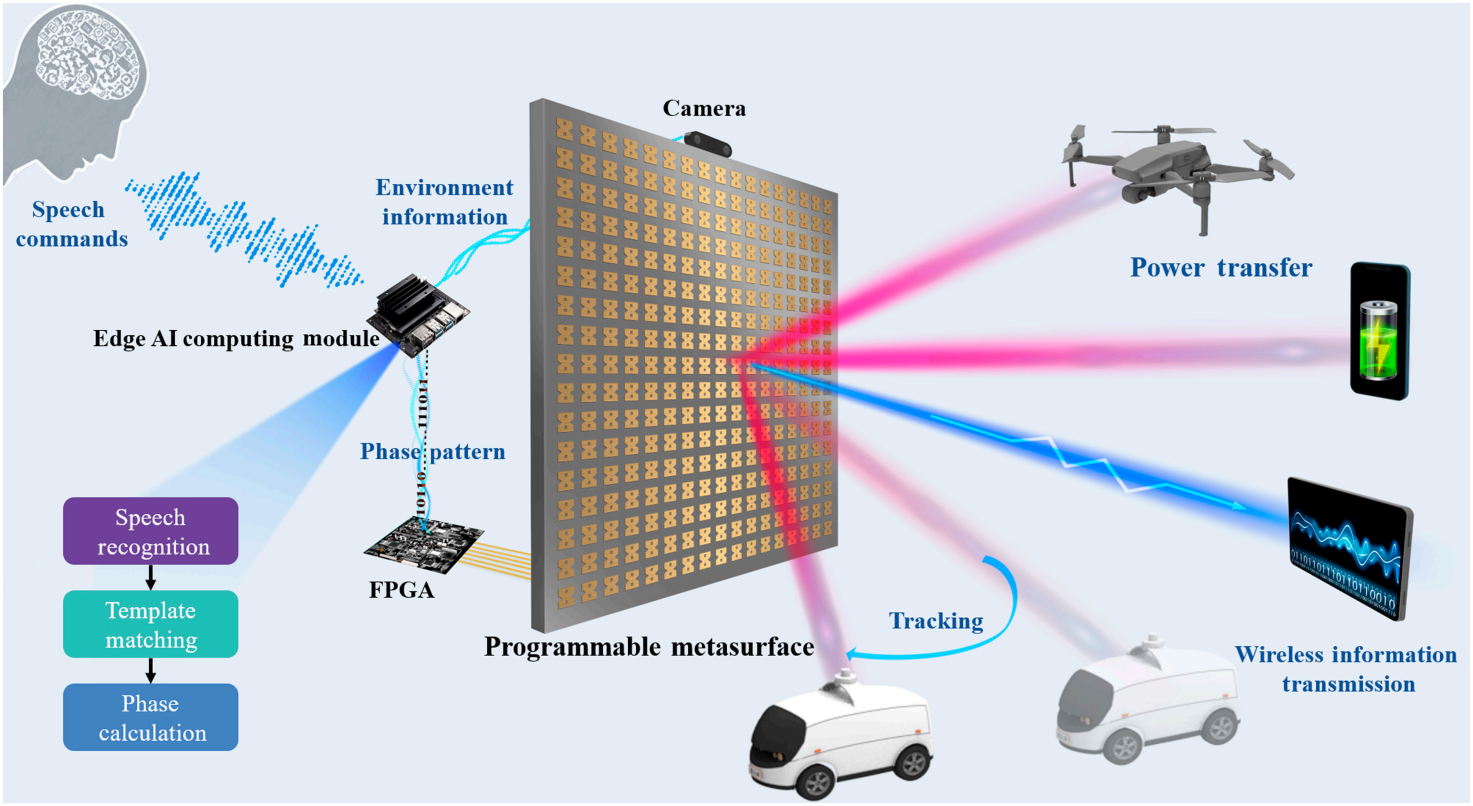
The framework of the proposed speech-controlled reconfigurable intelligent metasurface. The speech-controlled reconfigurable intelligent metasurface consists of a depth camera, a speech recognition module, an edge artificial intelligence (AI) computing module, a field-programmable gate array (FPGA), and a programmable metasurface. Based on speech commands, the system enables wireless power transfer and wireless information transmission to multiple targets in dynamic and complex environments.

Figure 1 shows the proposed SC-RIS, which consists of a depth camera, a speech recognition module, a field-programmable gate array (FPGA), an edge artificial intelligence (AI) computing module, and a PMS. The depth camera is used to capture environmental information and generate both image and location data. The edge AI computing module processes these data using a template matching algorithm to recognize charging and information transmission targets while ensuring real-time performance and energy efficiency. After receiving speech commands through the speech recognition module, the edge AI computing module calculates the phase pattern through NFF beamforming and transmits it to the FPGA, which then controls the PMS to enable wireless power and information transmission. In this way, the proposed SC-RIS forms a self-adaptive closed-loop control system that operates in speech-command mode, enabling wireless charging and information transmission to static or dynamic targets while eliminating the need for manual operation or physical contact. The following sections discuss the design and implementation of the RIS system.

**Fig. 2.**
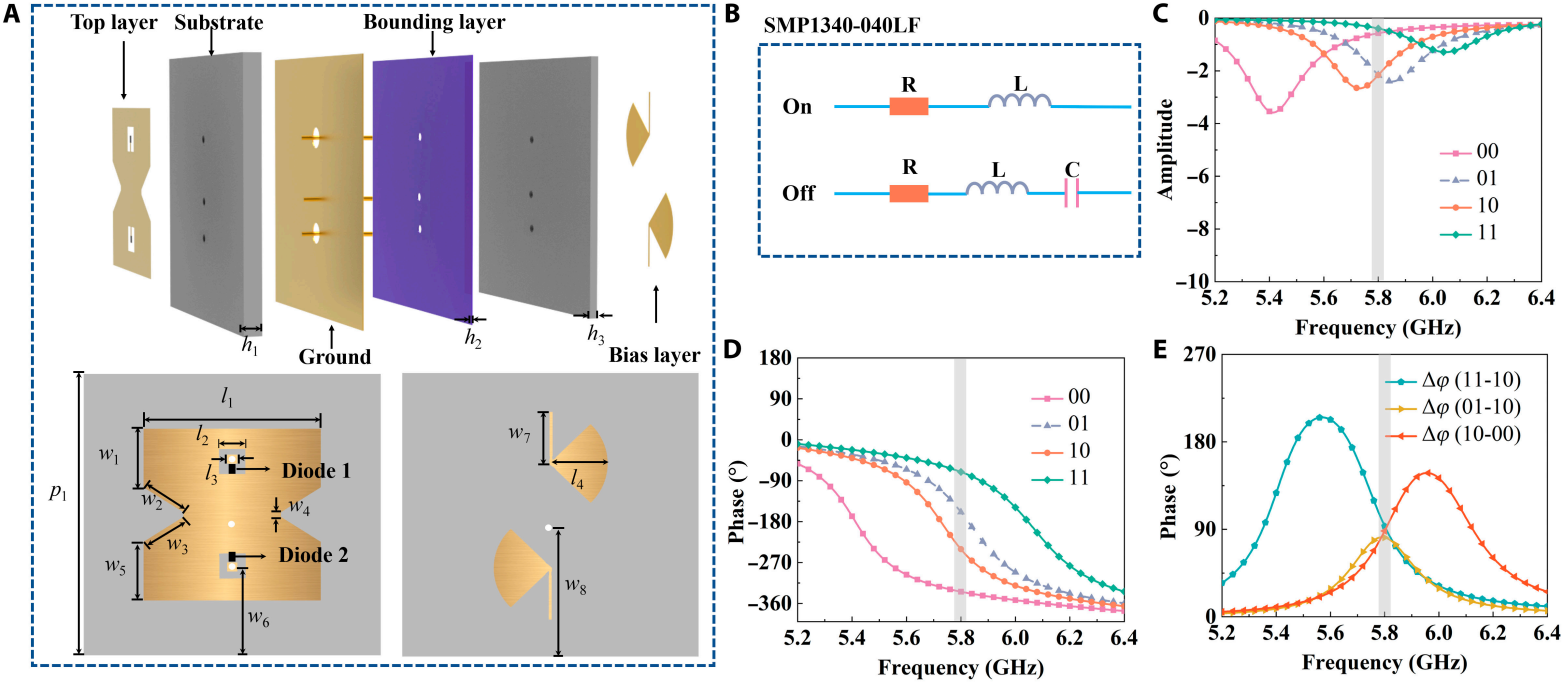
Schematic and performance characterization of the proposed meta-atom. (A) Exploded and top views of both top and bottom metal layers. (B) Equivalent circuit of the integrated PIN diode. (C to E) Simulated results for reflective amplitude, phase, and phase difference under different diode switching states. *h*_1_ = 1.51 mm, *h*_2_ = 0.10 mm, *h*_3_ = 0.69 mm, *l*_1_ = 13.10 mm, *l*_2_ = 2.00 mm, *l*_3_ = 0.60 mm, *l*_4_ = 4.30 mm, *w*_1_ = 4.55 mm, *w*_2_ = *w*_3_ = 3.54 mm, *w*_4_ = 0.40 mm, *w*_5_ = 4.55 mm, *w*_6_ = 7.80 mm, *w*_7_ = 4.00 mm, *w*_8_ = 10.40 mm, and *p*_1_ = 22.00 mm.

The meta-atom consists of 3 copper layers (top, ground, and bottom), each with a thickness of 0.035 mm, as well as 3 dielectric substrates with thicknesses *h*_1_, *h*_2_, and *h*_3_, as shown in Fig. 2A. The top layer utilizes a metal hourglass-shaped structure with 2 PIN diodes (Skyworks, SMP1340-040LF) aligned in opposite directions. For the SMP1340-040LF PIN diode, a forward bias voltage of approximately 0.65 V is required for switching. The device exhibits a typical switching time of around 10 ns, making it suitable for high-speed RF applications. The ground is connected to the top layer through the middle metal vias. The bias layer composed of 2 sector-shaped structures isolates high-frequency signals and connects to the top layer through 2 dedicated vias. The 2 dielectric substrates on each side are made of F4B ($\varepsilon_{r}=2.65$ and tanδ=0.0015), and the bounding layer is made of Rogers RO4450F ($\varepsilon_{r}=3.52$ and tanδ=0.004). By introducing 2 dedicated vias positioned at different distances from the central metal via, the meta-atom operates similarly to an EM bandgap structure. The switching states of the PIN diodes modify the metal hourglass-shaped structure, thereby altering its resonant frequency. Specifically, the switching changes the current path through the hourglass-shaped geometry, resulting in different resonant modes and corresponding phase shifts. Consequently, the meta-atom can generate distinct phase shifts under different biasing conditions. The meta-atom is simulated using CST Microwave Studio 2020, with periodic boundary conditions applied along the *x* and *y* directions. Due to the limitations of the meta-atom surface structure, the proposed metasurface can operate only under *y*-polarized incidence. Therefore, a *y*-polarized EM wave incident along the −z direction is used as the excitation source. The state “01” is defined as diode 1 being turned off and diode 2 being turned on, and similarly, the other 3 states are defined accordingly. The equivalent circuit model of the PIN diode adopts a 1-Ω resistor in series with a 0.45-nH package inductor to represent the on state, while the off state is characterized by a 10-Ω resistor in series with a 0.45-nH inductor and a 0.16-pF capacitor, as shown in Fig. 2B. Figure 2C illustrates the reflective amplitude for the 4 states of the meta-atom. The results indicate that the reflective amplitude for all 4 states is above −2.2 dB, demonstrating efficient reflection of EM waves at 5.8 GHz. Figure 2D illustrates the reflective phase response of the proposed meta-atom in its 4 states. The results show that the 4 states exhibit a significant equal-phase difference at 5.8 GHz. As shown in Fig. 2E, the reflective phase difference Δϕ between the 4 states remains close to 90° at 5.8 GHz, allowing for 2-bit phase modulation. To analyze the performance of the proposed meta-atom under different incident angles, simulations were performed at various incident angles, as shown in Note S1. The results show that the incident angle should not exceed 45° to ensure optimal performance.

The SC-RIS adopts NFF beamforming to concentrate power on static or dynamic targets. A horn antenna operating at 5.8 GHz is used to radiate the metasurface as a feed source. At the center of each meta-atom, the tangential electric field $E_{r}\left(x_{i},y_{j}\right)$ is formulated as

$$\begin{array}{c} \begin{array}{c} E_{r}\left(x_{i},\;y_{j}\right)=A_{\mathrm{ij}}\text{exp}\left(j\phi_{\mathrm{ij}}\right)\cdot E_{0}\left(x_{i},\;y_{j}\right)\\ \;\cdot \text{exp}\left(jk_{1}\sqrt{{\left(x_{s}-x_{i}\right)}^{2}+{\left(y_{s}-y_{j}\right)}^{2}+z_{s}^{2}}\right) \end{array} \end{array}$$ (1)

where $\left(x_{s},\;y_{s},\;z_{s}\right)$ denotes the spatial position of the feed source’s phase center, *E*_*0*_(*x*_*i*_*,y*_j_) is tangential E-field incident from the feed source, $A_{\mathrm{ij}}$ and $\phi_{\mathrm{ij}}$ denote the reflection coefficient magnitude and phase of the $\left(i,j\right)\mathrm{th}$ meta-atom, respectively, while $k_{1}$ denotes the wave number at 5.8 GHz. Once the desired group of focal points is determined as $\sum_{l=1}^{L}\left(x_{f_{i}},\;y_{f_{i}},\;z_{f_{i}}\right)$, the corresponding $\phi_{\mathrm{ij}}$ can be calculated by [55]

$$\begin{array}{l} \phi_{\mathrm{ij}}=-k_{1}\sqrt{{\left(x_{s}-x_{i}\right)}^{2}+{\left(y_{s}-y_{j}\right)}^{2}+z_{s}^{2}}\\ \;-\text{arg}\left\{\sum_{l=1}^{L}\text{exp}\left(jk_{1}\sqrt{{\left(x_{f_{i}}-x_{i}\right)}^{2}+{\left(y_{f_{i}}-y_{j}\right)}^{2}+z_{f_{i}}^{2}}\right)\right\} \end{array}$$ (2)

Owing to the 2-bit quantization of the meta-atom phase states, the reflection phase $\phi_{\mathrm{ij}}$ needs to be quantized as follows:

$$\phi_{\mathrm{ij}}^{q}=\left\lfloor \frac{\phi_{\mathrm{ij}}}{\pi /2}\right\rfloor \cdot \frac{\pi }{2}$$ (3)

where $\phi_{\mathrm{ij}}^{q}$ represents the quantized phase and $\left\lfloor *\right\rfloor$ denotes the rounding operation. The array coding matrix can be determined using the above equations.

**Fig. 3.**
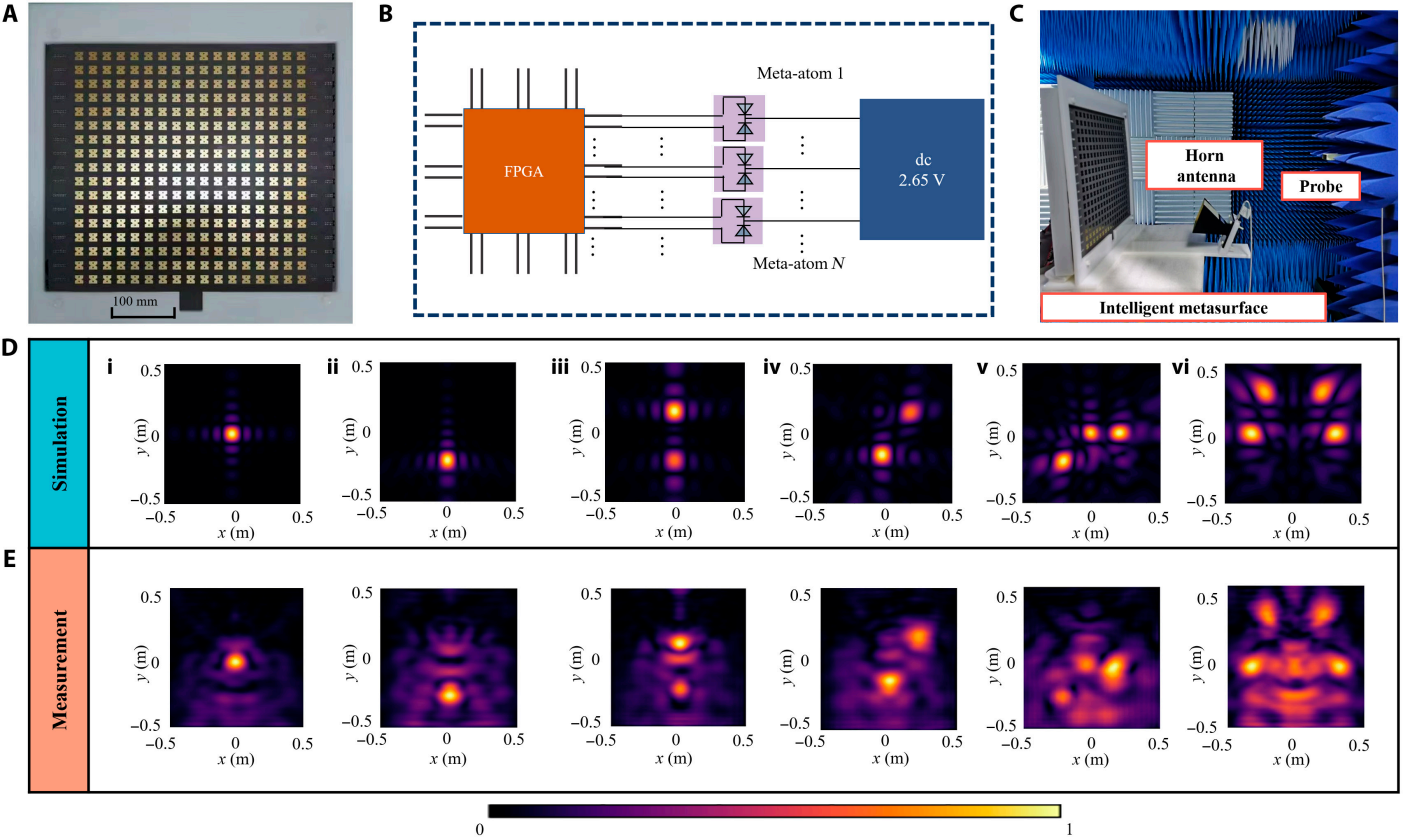
Measurements of the prototype reconfigurable intelligent metasurface. (A) A photograph of the prototype reconfigurable intelligent metasurface. (B) Schematic diagram of the FPGA control circuit. (C) Experimental environment of near-field focusing. (D) Simulated electric-field intensity distributions for different focal configurations from left to right: (i) single-focus, (ii) single-focus, (iii) dual-focus case 1, (iv) dual-focus case 2, (v) triple-focus, and (vi) multifocus. (E) Measured electric-field intensity distributions corresponding to the same focal configurations as in (D).

To achieve dynamic multiple-focus NFF, a prototype RIS with 17 × 17 (289) meta-atoms is fabricated, as illustrated in Fig. 3A. Figure 3B plots the control circuit of the RIS. Each PIN diode in the meta-atom is connected to an independent serial port of the FPGA via a dedicated feed line drawn from the bias layer. Additionally, a 2.65-V dc power supply is connected to the ground plane of the SC-RIS. The states of each meta-atom can be switched by changing the FPGA’s input voltage (0/3.3 V). Figure 3C shows the near-field experiment environment for measuring the NFF of the RIS. A planar near-field scanning system is employed to measure the spatial distribution of the electric-field intensity. A rectangular waveguide probe is used to scan a 1.00 × 1.00 m square plane. For each focal configuration, the scanning plane was positioned at a distance from the metasurface corresponding to the *z* coordinate of the focal points, ensuring accurate field measurements at the respective focal planes. The metasurface is fed by an obliquely incident horn antenna to avoid the blockage effect of the feed horn. The horn antenna operates over the 4.64- to 7.05-GHz frequency range with a gain of 15 dB, consistent with the horn antenna used in the simulation setup. The feeding horn is positioned 390 mm away from the metasurface with an incident angle of 22.5°, and its phase center is located at (0.00, −0.11, 0.41) m relative to the center of the metasurface. Figure 3D and E illustrate the simulated and experimental results of electric-field distributions corresponding to single-focus and multifocus spot configurations. To examine the control flexibility of the RIS, 6 NFF electric-field intensity distributions are simulated and measured: 2 single-focal spots located at (0.00, 0.00, 0.80) m and (0.00, −0.30, 0.80) m; 2 dual-focal spot cases: case 1, (0.00, 0.20, 1.00) m and (0.00, −0.20, 1.00) m, case 2, (0.15, 0.20, 1.00) m and (0.00, −0.20, 1.00) m; 3 focal spots at (0.00, 0.00, 1.00) m, (0.15, 0.00, 1.00) m, and (−0.15, −0.30, 1.00) m; and 4 focal spots at (0.40, 0.00, 1.00) m, (−0.40, 0.00, 1.00) m, (0.30, 0.30, 1.00) m, and (−0.30, 0.30, 1.00) m. The results show that all focal spots closely match their target positions, confirming that the metasurface enables programmable control for dynamic multifocus near-field applications.

**Fig. 4.**
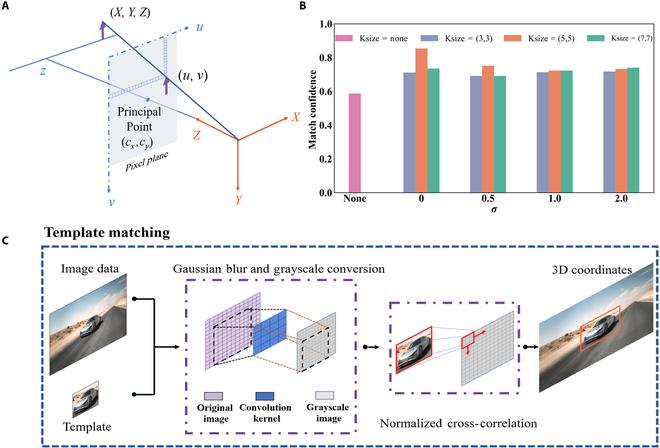
(A) The camera model, which encompasses both the camera coordinate system and the pixel coordinate system. (B) Simulation of matching confidence with different Gaussian blurs. (C) Structure diagram of the template matching algorithm. 3D, 3-dimensional.

The edge AI computing module is used to implement algorithms for target detection and control computation, with the aim of reducing system power consumption and improving system integration. Jetson Orin Nano 4 GB is an entry-level compact edge AI computing module in the NVIDIA Jetson Orin series. It can provide up to 20 Tera Operations Per Secon (TOPS) of AI inference performance under 10 W of power consumption, making it well suited for resource-constrained edge applications such as entry-level robots, smart cameras, and low-power vision systems. In our system, it handles template matching and phase computation tasks that involve complex floating-point operations and real-time data processing, which are difficult to implement efficiently on the FPGA. Target detection is primarily achieved using a depth camera and the template matching algorithm. The depth camera (Astra Pro Plus) is used to collect environmental information in real time at a rate of 30 fps. To convert pixel coordinates into 3-dimensional (3D) coordinates in the camera, a camera model is established that bridges the pixel and camera coordinate systems, as illustrated in Fig. 4A. The corresponding 3D coordinates in the camera can be calculated by

$$X=\frac{\left(u-c_{x}\right)\cdot z}{f_{x}}$$ (4)

$$Y=\frac{\left(v-c_{y}\right)\cdot z}{f_{y}}$$ (5)

Z=z (6)

where u and v represent the coordinates of the image pixel; z is the corresponding depth value of the pixel; X, Y, and Z are the 3D coordinates in the camera coordinate system; $f_{x}$ and $f_{y}$ denote the focal lengths of the camera; and $c_{x}$ and $c_{y}$ are the coordinates of the camera’s principal point. $f_{x}$, $f_{y}$, $c_{x}$, and $c_{y}$ are intrinsic to the camera and are typically obtained through a standard camera calibration process. The pixel coordinates (u, v) are directly extracted from the 2-dimensional image captured by the camera, specifying the column and row indices of each pixel. In depth camera systems, the depth value z is retrieved from the corresponding depth map, in which each pixel stores a scalar value indicating the distance from the camera to the surface point. The 3D coordinates of the targets can be obtained by the above equations.

Figure 4C shows the structural diagram of the template matching algorithm. The template matching algorithm is used primarily to locate regions within an image that match a given template and is applied to process the captured image data. To improve matching confidence, Gaussian blur and grayscale conversion are applied as preprocessing steps in the template matching algorithm. Gaussian blur and grayscale conversion help reduce image noise, smooth fine details, and enhance contour stability, thereby reducing computational complexity while preserving the main structural information. Gaussian blur is performed by convolving the original image with a pre-defined Gaussian kernel to produce a blurred image. The Gaussian kernel is defined as

$$G\left(x_{g},\;y_{g}\right)=\frac{1}{2{\pi \sigma }^{2}}e^{-\frac{{x_{g}}^{2}+{y_{g}}^{2}}{2\sigma^{2}}}$$ (7)

where $x_{g}$ and $y_{g}$ denote the horizontal and vertical distances from the center of the filter kernel, respectively, and σ represents the standard deviation, which controls the degree of blurring: the higher the value, the stronger the blur. The matching confidence of the car image in Fig. 4C is simulated under different levels of Gaussian blur, as shown in Fig. 4B. Compared with the case without Gaussian blur, the matching with Gaussian blur is improved by at least 17.78%. Under the condition of kernel size (5,5) and σ=0, the matching confidence reaches 85.45%. The Gaussian kernel is finally set to Ksize = (5,5) and σ=0 for image preprocessing in the template matching algorithm. The matching method in the template matching algorithm adopts normalized cross-correlation (NCC), which can be expressed as

$$\mathrm{NCC}\left(x_{n},\;y_{n}\right)=\frac{\sum_{i,j}\left(T\left(i,\;j\right)-\bar{T}\right)\left(I\left(x_{n}+i,\;y_{n}+j\right)-\bar{I}\right)}{\sqrt{\sum_{i,j}{\left(T\left(i,\;j\right)-\bar{T}\right)}^{2}\sum_{i,j}{\left(I\left(x_{n}+i,\;y_{n}+j\right)-\bar{I}\right)}^{2}}}$$ (8)

where $x_{n}$ and $y_{n}$ denote the top left coordinates of the sliding window in the image, i and j are the pixel coordinates in the template, $T\left(i,\;j\right)$ is the gray value of the template at pixel $\left(i,\;j\right)$, I represents the image region to be matched, $\bar{T}$ is the average gray value of the template, and $\bar{I}$ is the average gray value of the current sliding window in the image. The threshold for matching confidence is set between 0.7 and 1 to improve the sensitivity of target detection and to allocate computing resources more effectively. The control computation algorithm consists of 2 parts: speech recognition and phase calculation. The speech commands are recognized by the speech recognition module (WonderEcho), which captures the voice input and transmits the corresponding binary data to the edge AI computing module by I^2^C communication. The phase calculation is based on NFF beamforming and is accelerated by graphics processing unit parallel computing on the edge AI computing module to achieve real-time performance and fast response. The FPGA receives commands from the edge AI computing module via serial communication to control the PMS, which generates dynamic NFF. The power consumption of the SC-RIS control system can be divided into 3 main components. The depth camera and speech recognition module are powered by the edge AI computing module, while the FPGA and a 2.65-V dc power supply are used to drive the PMS. The power consumption of the edge AI computing module averages 6.6 W during operation, with a peak value of 6.8 W as measured using the system monitoring tool (JTOP). The metasurface is controlled in parallel by 3 FPGAs (XC7K325T), with a combined power consumption of 3 W, measured using an electric energy meter. The 2.65-V dc power supply consumes 0.04 W. Therefore, the total power consumption of the SC-RIS is 9.84 W, demonstrating its low-power advantage and suitability for energy-constrained applications in the IoT.

### Experimental verification

**Fig. 5.**
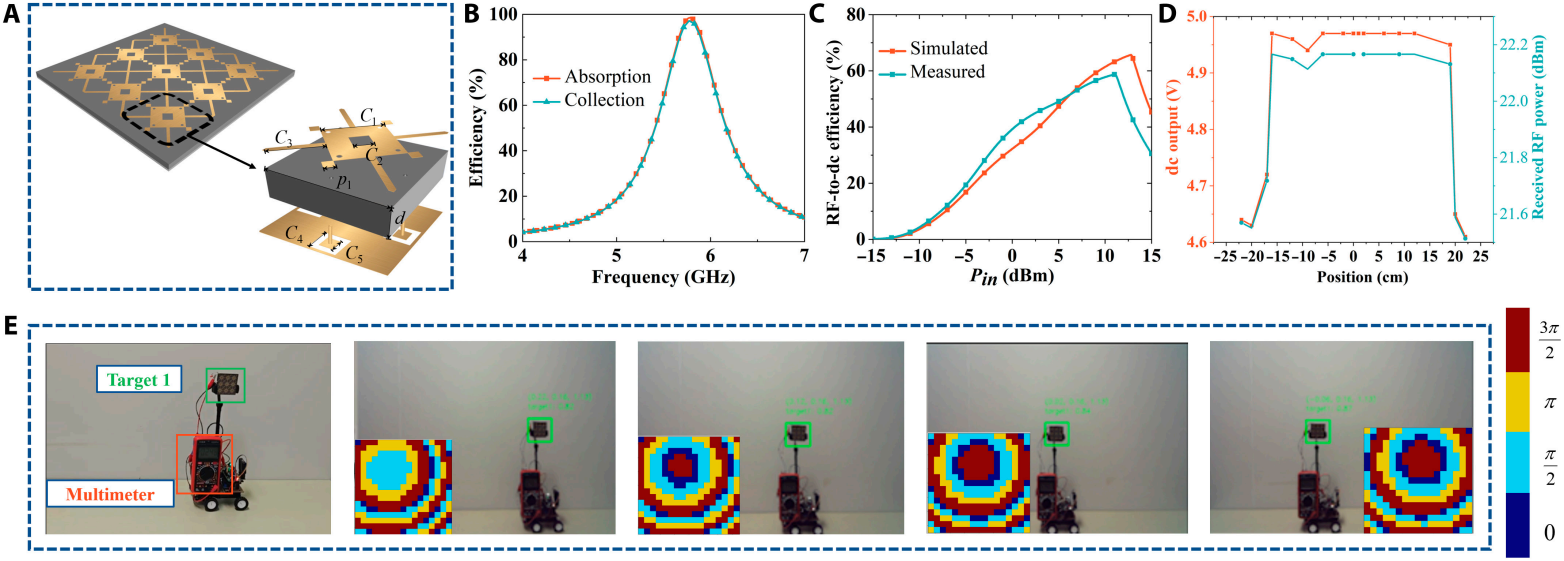
Schematic diagram of the proposed rectifying metasurface and experimental results of dynamic-target wireless power transfer. (A) Exploded view of the rectifying metasurface meta-atom. (B) Simulated absorption and collection efficiency of the rectifying metasurface. (C) Simulated and measured RF-to-dc conversion efficiency of the rectifying metasurface. (D) Measured results of the received RF power and dc output. (E) Experiment on dynamic-target wireless power transfer. *C*_1_ = 11.60 mm, *C*_2_ = 3.60 mm, *C*_3_ = 9.33 mm, *C*_4_ = 3.40 mm, *C*_5_ = 1.40 mm, and *P*_1_ = 22.10 mm.

A rectifying metasurface (RMS) is proposed to efficiently receive and convert the focused energy generated by the SC-RIS into a dc output as shown in Fig. 5A. The proposed RMS consists of 9 meta-atoms, a low-pass filter, a 150-Ω load, and a lamp. Figure 5A presents the exploded view of the meta-atom, comprising two 0.035-mm-thick copper layers and a dielectric substrate of thickness *d* = 3 mm. The top structure consists of 4 square patches and a square-shaped ring, forming a rotationally symmetric geometry that ensures polarization-insensitive performance, thereby enhancing EM energy absorption. The meta-atoms are connected by 4 dc bias lines, enabling energy transfer to a low-pass filter and a load. Four Schottky diodes (HSMS-2860) are placed on the ground and are connected to the top structure through 4 metalized vias to rectify the absorbed EM energy. The placement of the 4 Schottky diodes can efficiently minimize the structural impact caused by diode soldering. The simulated absorption and collection efficiencies of the RMS are shown in Fig. 5B. The absorption represents the RMS’s ability to absorb EM energy. The collection efficiency is the ratio of the transmitted energy of the rectifier diode to the energy absorbed by the RMS. These metrics can efficiently analyze the performance of the RMS. According to the results, the RMS demonstrates an absorption of 98.50% and a collection efficiency of 97.20% at 5.8 GHz, which means that it can efficiently absorb the energy transmitted by the RIS and deliver it to the rectifier diode. Figure 5C shows the simulated and measured RF-to-dc efficiency of the RMS, which achieves the highest efficiency of 58.73% with an input power of 11 dBm at 5.8 GHz.

Furthermore, to verify the dynamic-target WPT capability of the RIS, the dc output and received RF power of the RMS are measured. The experimental setup for dynamic-target WPT is shown in Fig. 5E. The multimeter is connected in parallel to the load to measure the voltage and placed in the electric vehicle. The RMS is placed at a distance of *y* = 0.14 m and *z* = 1.13 m from the center of the RIS, while the value of *x* changes with the movement of the electric vehicle. The SC-RIS is excited by a horn antenna, which is connected to a 5.8-GHz signal generator with an RF output power of 40 W. When the RIS receives the speech command, it focuses wireless power on the moving target (the RMS). The RMS receives the RF energy and converts it into dc energy, and the multimeter displays the voltage throughout the load. The detailed experimental process can be seen in Movie S1. The measured results of the received RF power reception and dc output are shown in Fig. 5D. It can be seen from the results that the received RF power and dc output change as the electric vehicle moves, but the received RF power remains above 25.13 dBm and the dc output remains above 4.61 V, demonstrating that the proposed RIS can achieve stable and real-time wireless energy transfer to dynamic targets. However, it should be noted that while the system is suitable for low-power applications, its ability to support high-power wireless energy transfer is still limited and requires further improvement in future work.

**Fig. 6.**
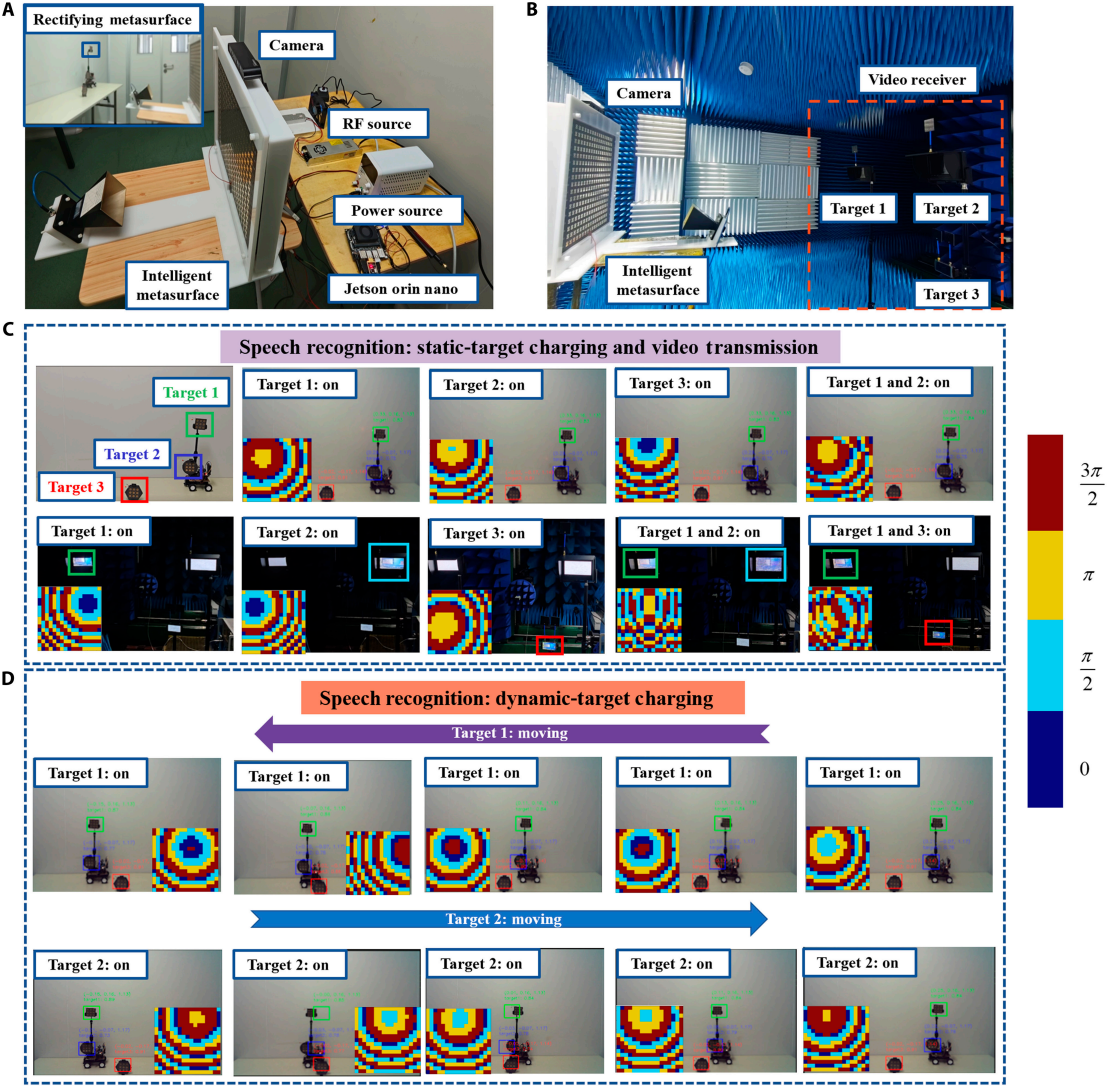
Wireless power transfer (WPT) and video transmission experiments. Experimental setup for (A) WPT and (B) video transmission. (C) The experiment for static-target WPT and video transmission controlled by speech commands. (D) The experiment for dynamic-target WPT and video transmission controlled by speech commands.

The proposed SC-RIS is experimentally demonstrated to be capable of intelligently delivering wireless power and video transmission to desired targets, as shown in Fig. 6. The experiments are divided into WPT and information transmission, and the experimental setups are shown in Fig. 6A and B. Three targets (denoted as a rectangle, a circle, and a hexagonal RMS with a lamp) are placed on an electric vehicle and a table in the WPT experiment. The horn antenna is connected to a 5.8-GHz signal generator with an RF output power of 40 W. In the information transmission experiment, these targets are replaced by 3 video receivers. The horn antenna is connected to the video transmission module (TS5823 pro). In the first experiment, the proposed SC-RIS demonstrated the ability to selectively charge or transmit video to static targets based on speech commands, as shown in Fig. 6C (see also Movies S2 and S3). In this experiment, the SC-RIS obtains the spatial position information of targets through the camera. The SC-RIS will then transmit power or video to a single static target or multiple targets according to the received speech commands. Subsequently, the corresponding lamp on the RMS or the video receiver at the static single target or multiple targets will be turned on. In the second experiment, the proposed SC-RIS demonstrated the ability to selectively charge dynamic targets based on speech commands, as shown in Fig. 6D. Unlike in the first experiment, target 1 starts moving from right to left at a speed of 0.06 m/s. When the SC-RIS receives the speech command to charge target 1, it charges target 1 in real time based on the changing spatial position information, and the corresponding lamp on the RMS turns on. Next, the speech command changes to charging target 2 as the electric vehicle moves to the left edge of the table. Subsequently, target 1 is turned off, and target 2 is turned on. Target 2 remains on as the electric vehicle moves back to the starting point. Moreover, in practical environments, interference from other communication devices operating in adjacent frequency bands can arise. The programmable capabilities of the SC-RIS itself, along with other potential approaches, can help eliminate such interference (see Note S2 for details). Meanwhile, to measure the response time of dynamic-target tracking, the time module was introduced for timing. The results show that the response time does not exceed 0.06 s (see Note S3 for details). The experiments sufficiently demonstrate that the SC-RIS can selectively enable wireless charging and information transmission to static or dynamic targets based on speech commands, thereby eliminating the need for manual operation or physical contact. The detailed experimental process can be seen in Movies S2 and S3. A comparison between the proposed SC-RIS and similar RIS designs reported in the literature is presented in Note S4. The comparison indicates that the proposed SC-RIS, featuring voice-interactive control and system power consumption below 10 W, is well suited for practical applications requiring low power and real-time user interaction.

## Conclusion

In this paper, we propose an SC-RIS system, which consists of a camera, a speech recognition module, an edge AI computing module, an FPGA, and a PMS. The proposed RIS can achieve real-time data communication and efficient WPT to static or moving targets by combining speech recognition, computer vision, target detection, and dynamic beam control. To reduce power consumption and improve system integration, the template matching algorithm and the control computation algorithm were deployed in the edge AI computing module, resulting in a total power consumption of approximately 9.84 W for the SC-RIS. Unlike conventional RISs that rely on pre-defined control and external processing units, the proposed scheme equips the metasurface with visual and linguistic perception capabilities, thereby enabling a transition from passive reconfiguration to actively adaptive multimodal intelligence. Experimental results confirm that the proposed SC-RIS system provides a stable dc output exceeding 4.61 V on dynamic targets, which is sufficient to supply power to small IoT devices such as sensors, microcontrollers, and medical sensors for brain–computer interface applications. It also enables flexible point-to-point WPT and communication through touchless speech interaction. The proposed SC-RIS demonstrates significant potential for diverse applications in the IoT, including sensors, small mobile devices, smart home systems, and unmanned aerial vehicles.

## Methods

Details about the simulation and experimental methods used in this study, including the meta-atom simulation in CST Microwave Studio, the configuration of near-field focusing under different interference scenarios, the response time of the dynamic target tracking, and the comparison of the proposed SC-RIS with existing RIS systems, are fully described in the Supplementary Materials (Notes S1 to S4 and Figs. S1 to S4).

## Data Availability

The data that support the findings of this study are available from the corresponding authors upon reasonable request.

## Supplementary Materials

Supplementary 1 Notes S1 to S4 Figs. S1 to S5 Table S1 Movies S1 to S3
