# Supplementary material for: Speech-Controlled Reconfigurable Intelligent Metasurface for Real-Time Wireless Power Transfer and Communication
Source: Research (Wash D C). 2025 Aug 12;8:0831. doi: 10.34133/research.0831 (PMC12340224; doi:10.34133/research.0831)
Supplement: Supplementary 1 — Notes S1 to S4 Figs. S1 to S5 Table S1 Movies S1 to S3 [file research.0831.f1.zip › Supporting Information.docx]

**Supplementary Materials for**

**Speech-Controlled Reconfigurable Intelligent Metasurface for Real-Time Wireless Power Transfer and Communication**

Lin Dong^1^, Liming Si^1*^, Yueze Liu^2^, Qitao Shen^1^, Pengcheng Tang^1^, Genhao Wu^1^, Rong Niu^1^, Qingqing Wu^3^, and Weiren Zhu^3*^

^1^ Beijing Key Laboratory of Millimeter Wave and Terahertz Technology, School of Integrated Circuits and Electronics, Beijing Institute of Technology, Beijing 100081, China, State Key Laboratory of Environment Characteristics and Effects for Near-space, Beijing Institute of Technology, Beijing 100081, China

^2^ School of Mechanical Engineering, Beijing Institute of Technology, Beijing 100087, China

^3^ Department of Electronic Engineering, Shanghai Jiao Tong University, Shanghai 200240, China

*Address correspondence to: lms@bit.edu.cn

*Address correspondence to: [weiren.zhu@sjtu.edu.cn](mailto:weiren.zhu@sjtu.edu.cn)

**Supplementary Materials include:**

Supporting Note S1 to S4

Supporting Fig. S1 to S5

**Supporting Note S1**

**The Characterization of the Proposed meta-atom Under Different Incident Angles**

The meta-atom is simulated using CST Microwave Studio 2020 with periodic boundary conditions applied along the x- and y-directions. A y-polarized electromagnetic wave incident along the −z direction at different angles is used as the excitation source. As shown in Figure. S1, the amplitude of the meta-atom decreases as the incident angle increases. At an incident angle of 60°, the amplitude of the 01 state is -7 dB. Nonetheless, the amplitude of the meta-atom remains higher than -4 dB for incident angles ranging from 0° to 45°. The reflective phase variation with incident angle is similar to that of the amplitude. The reflective phase remains relatively stable, staying around 0°, 90°, 180°, and 270° for the corresponding states when the incident angle ranges from 0° to 45°.Therefore, the incident angle should not exceed 45° to ensure optimal performance.


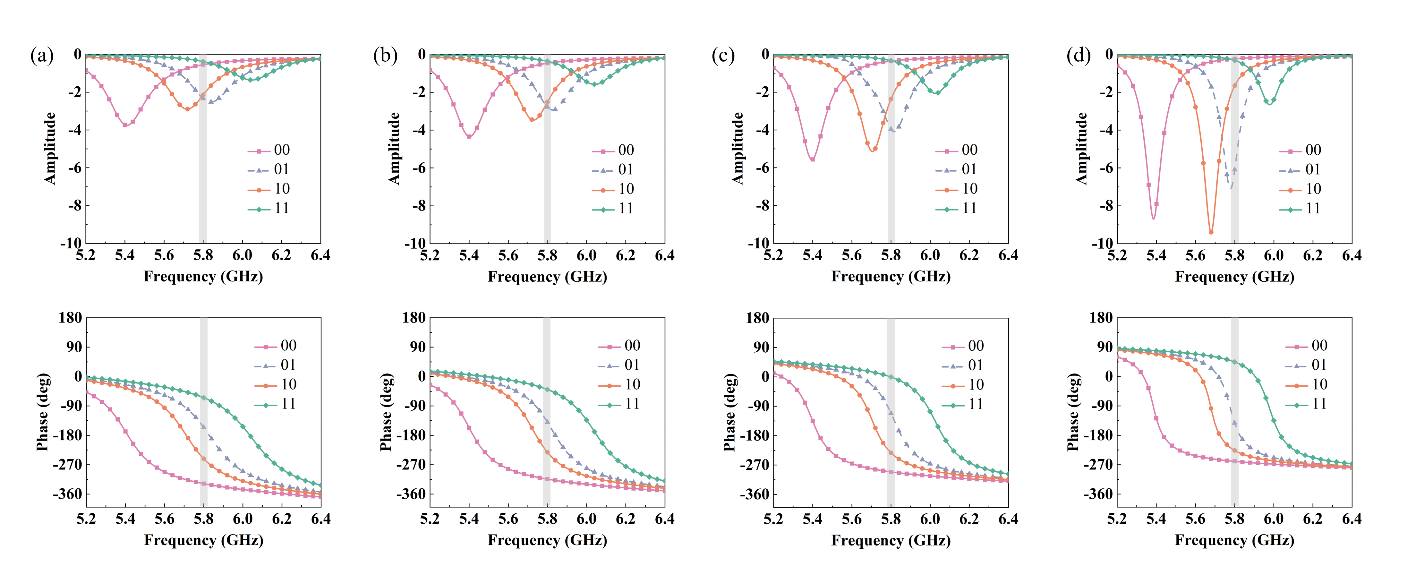


Figure S1: The simulated reflective amplitudes and phases. The incident angles: (a) 15°; (b) 30°; (c)45°; (d) 60°.

**Supporting Note S2**

**Discussion on how to solve the potential issue of interference from other actively communicating devices operating at a similar frequency**

In practical environments, the SC-RIS primarily faces electromagnetic interference and compatibility issues caused by other communication devices operating in adjacent frequency bands. To address these interferences, the following discussion will focus on the programmable capabilities of the SC-RIS itself.

Firstly, we demonstrate that the SC-RIS can effectively solve the potential issue of interference from other active communication devices operating at similar frequencies. The SC-RIS achieves near-field focusing by exploiting the phase differences between the four meta-atom states. As shown in Figure S1, the SC-RIS meta-atom exhibits its reflected phase response at 5.8 GHz, making it incapable of achieving near-field focusing for reflected electromagnetic waves at other frequencies.

Secondly, we demonstrate that the SC-RIS can suppress in-band interference from devices other than the feeding source. As shown in Figure S2, the coding pattern of the SC-RIS must be designed according to the phase center position of the feeding source to achieve phase compensation and near-field focusing.

Next, we investigate how increasing interference power affects system performance. In the commercial software CST, we set up a rectangular waveguide as the interference source, referred to as Port 2 (see Figure S3). Port 2 is positioned approximately 500 mm away from the DPM, with an incident angle of 0°. We carried out four simulation cases with the amplitude ratios of Port 1 to Port 2 set to 1:0, 0.5:1, 1:0.5, and 1:1, respectively, and the single focal spot positioned at (0.00, 0.00, 0.5) m. As shown in Fig. S4, the simulation results indicate that the power at the single focal spot located at (0.00, 0.00, 0.8) m is the highest among the four simulation cases. Meanwhile, we selected a circle with a radius of 50 mm centered at the focal point as the focal area, and calculated the focal power by integrating the Poynting vector within this region, as given by the following equation:

$$P=\int\int_{S}\left|S\right|dS$$

The focal efficiency is defined as the focal power divided by the total power of the feed source. The focal efficiency is 19.16% for the 1:0 case, 21.70% for the 0.5:1 case, 22.36% for the 1:0.5 case, and 24.52% for the 1:1 case. The results indicate that the interference source does not affect the near-field focusing performance, and the additional power from the interference source actually increases the power at the receiving focus.

Below is a discussion of possible methods for eliminating same-frequency interference. Similar-frequency interference is a common problem in many scenarios, such as weather radar systems, where it can distort radar variable estimations. Various methods have been proposed and studied by researchers to address this issue. For example: (1) installing an isolation board, and (2) designing specific properties of the transmitter or receiver to suppress interference.


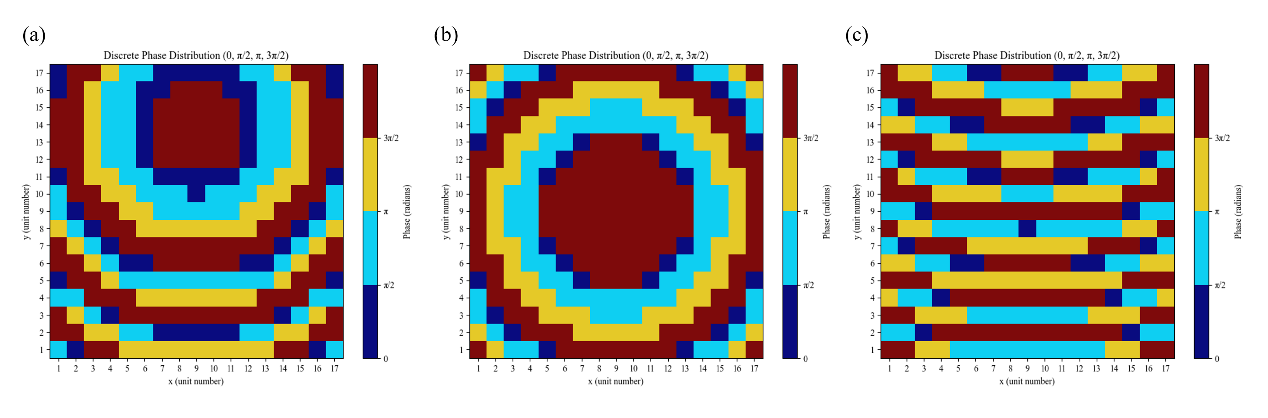


Figure S2: Different phase center positions of the horn antenna correspond to different SC-RIS coding patterns. (a) (0.00, -0.11, 0.41) m, (b) (0.00, 0.00, 0.41) m, (c) (0.00, -0.80, 0.41) m.


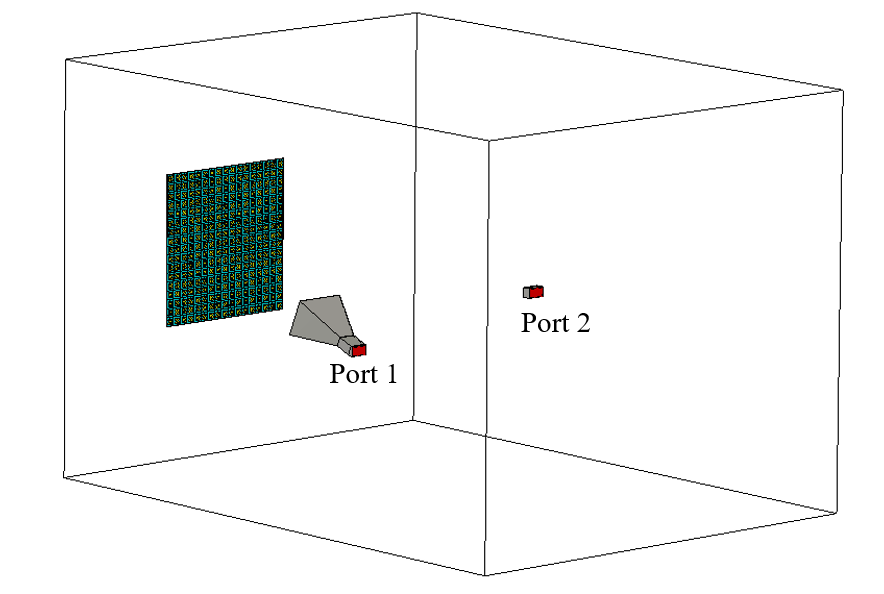


Figure S3: Simulation model of the proposed SC-RIS with two-port excitation.


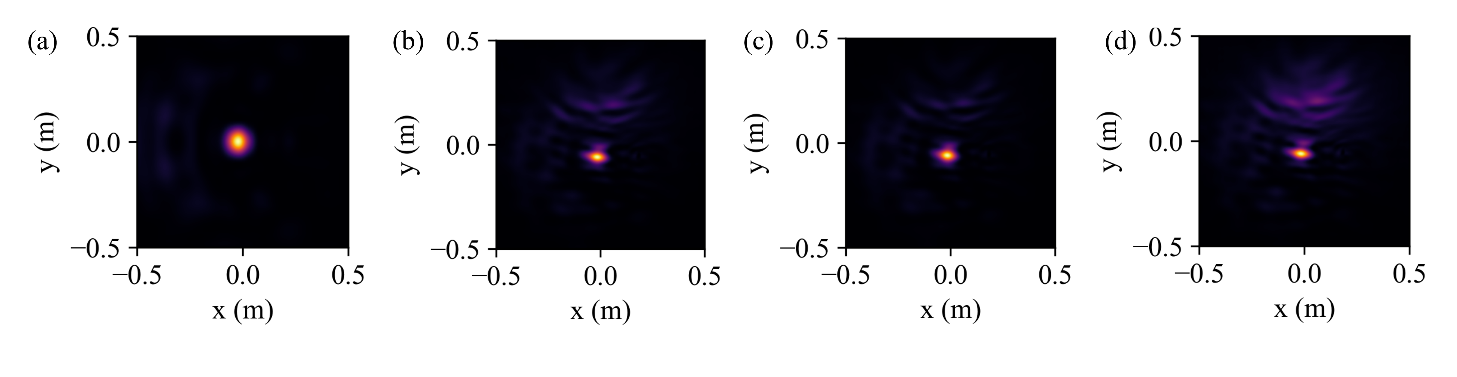


Figure S4: Near-field focusing results for four simulation cases with different amplitude ratios of Port 1 to Port 2: (a) 1:0, (b) 0.5:1, (c) 1:0.5, and (d) 1:1.

**Supporting Note S3**

**Discussion on the Speed of Dynamic Target Tracking and Its Response Time**

As shown in Supplementary Movies S2 and S3, the speed of the dynamic target is calculated based on the distance measured by the depth camera and the elapsed time, and is determined to be 0.06 m/s. The response time of dynamic target tracking includes the time required for the program to obtain the coordinates of the dynamic target, calculate the corresponding coding pattern, and transmit it to the metasurface. We introduce the time module to measure the response time of dynamic target tracking, as shown in Figure S5. The results show that the response time for dynamic target tracking does not exceed 0.06 s in any of the four cases.


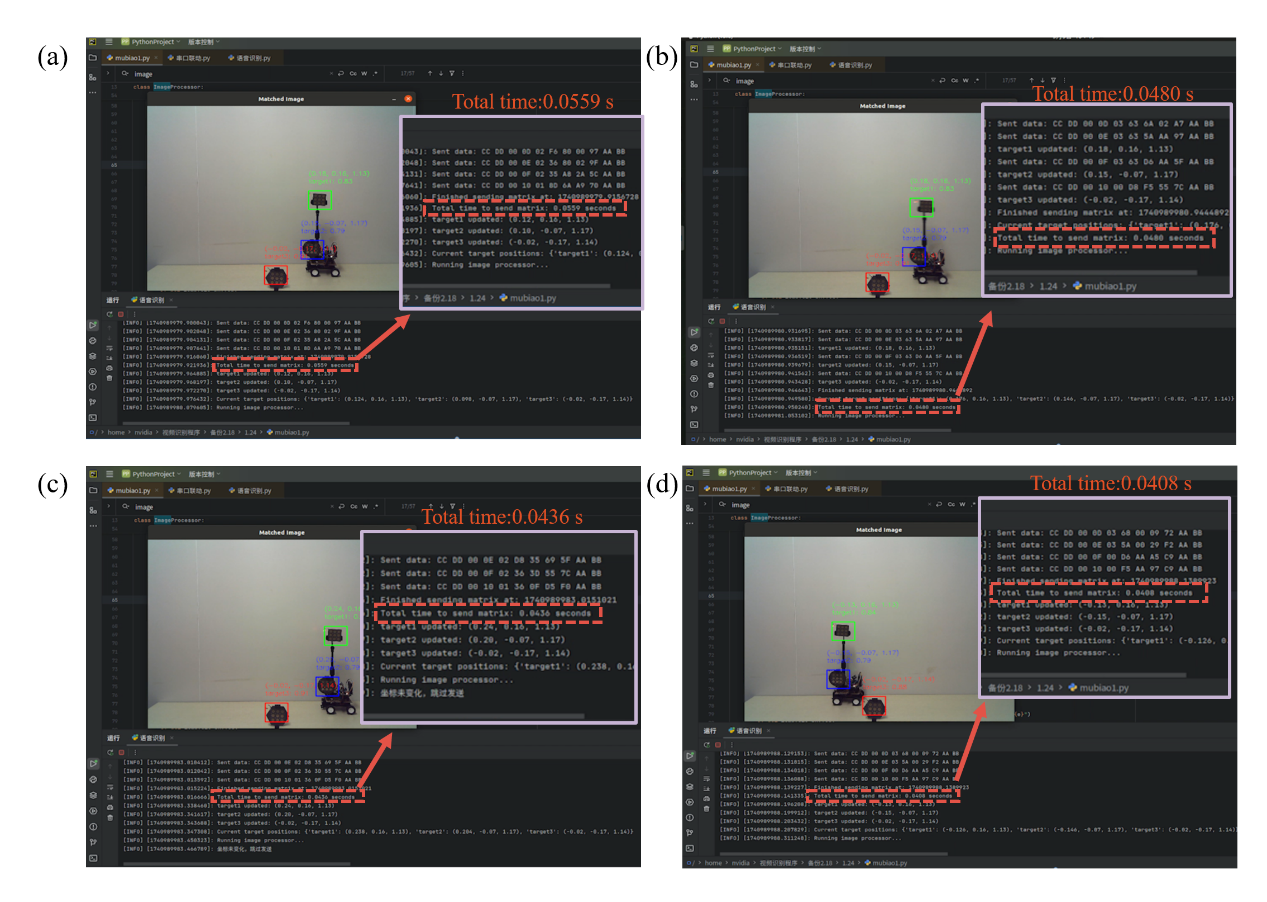


Figure S5: The response time of dynamic target tracking

**Supporting Note S4**

**Comparison between the Proposed SC-RIS and Similar RIS Designs Reported in the Literature**

Table S1 presents a comparison between the proposed SC-RIS and similar RIS designs reported in the literature. It can be seen that the RIS reported in the existing literature are mainly controlled through programming and lack responsiveness to changing user demands. Moreover, the control programs are generally deployed on laptops, resulting in system power consumption exceeding 10 W. In contrast, the proposed SC-RIS enables voice-interactive control of the metasurface and maintains system power consumption under 10 W. These advantages make the proposed SC-RIS more suitable for practical applications, especially in scenarios requiring low-power operation and real-time user interaction.

| Table.S1 Comparison between the Proposed SC-RIS and Similar RIS Designs Reported in the Literature | | | | | |
| --- | --- | --- | --- | --- | --- |
| Ref | Frequency | Interaction mode | Tracking mode | System power consumption | Function |
| [51] | 5.8 GHz and 11.6 GHz | Programming control | EM wave-based positioning | >10 W | Dynamic wireless energy transmission |
| [52] | 5.5~6.5 GHz | Voice interactive mode | Sensor-based positionin | \ | Static wireless energy and information transmission |
| [53] | 5.8 GHz | Programming control | vision-based positioning | >10 W | Dynamic wireless energy and information transmission |
| [54] | 5.8 GHz | Programming control | vision-based positioning | Standby:21.13 W  Running:40.13 W | Dynamic wireless energy and information transmission |
| This work | 5.8 GHz | Voice interactive mode | vision-based positioning | Running: 9.84 W | Dynamic wireless energy and information transmission |
